# Comparison of Efficacy in Improving Cardiopulmonary Resuscitation Performance between Video Training and the Standard Method

**Published:** 2018-07

**Authors:** Hamed Tavan, Ali Sahebi, Mohamad Golitaleb

**Affiliations:** 1 Department of Nursing, Faculty of Nursing and Midwifery, Student Research Committee, Ilam University of Medical Sciences, Ilam, Iran. 69391-77143. Tel: +98 841 2227123. Fax: +98 841 2227134. E-mail: hamedtavan@gmail.com.; 2 Department of Health in Disasters and Emergencies, School of Health, Safety and Environment, Shahid Beheshti University of Medical Sciences, Shahid Mostafa Khomaini Hospital, Ilam, Iran. 6931934833. Tel: +98 8433338228. Fax: +98 8433338455. E-mail: nurse.sahebi@yahoo.com.; 3 Department of Nursing, Arak University of Medical Sciences, Arak, Iran. 6941-7-38481. Tel: +98 8634173503-9. Fax: +98 8634173524. E-mail: m.golitaleb@arakmu.ac.ir.

Cardiopulmonary resuscitation (CPR) consists of a series of emergency procedures performed to save patients with urgent problems from death.^1^^, ^^2^ Given the rise in the prevalence of cardiovascular disease, it is vital that sufficient attention be paid to this group of patients.^3^

In the current study, initially, a CPR standard checklist containing 20 questions was scored on a 5-point Likert scale (from “completely comply with”=5 points to “not completely comply with”=1 point).^3^^, ^^4^ The total score of the CPR standard check list was 100 points, classified into 3 categories: weak (25–50points), moderate (51–75 points), and good (76–100 points). The sample size was calculated as 20 individuals in each group of case and control, comprised of nurses and doctors. The case and control groups were selected by identifying the number of the individuals and then matching them in terms of sex, work experience, and age. Consequently, 10 men and 10 women were assigned to each group. 

First, in a single-blind experiment, the researcher at the time of CPR supervised the performance of the study nurses and doctors and assigned scores to their performance based on the checklist. After CPR, training classes were held (only for the case group). In the classes, all the necessary measures considered useful during CPR such as massage administration, medication, intubation, and electroshock device application were introduced. Moreover, the case group received practical training face-to-face on how to set up and use the electroshock devices. The training videos were prepared on the basis of the participants’ response to the checklist. A copy of the checklist was given to the participants. After the class, the researcher re-evaluated the CPR performance of the case group based on the checklist.

After data collection, for ethical considerations, the control group was also provided with the training CD.

With regard to pre-training CPR performance, the findings showed that in the control group, 35% of the participants were weak (25–50 points), 60% were moderate (51–75 points), and 5%were good (76–100 points), whereas in the case group, 30% of the participants were weak (25–50 points), 65% were moderate (51–75 points), and 5%were good (76–100 points). Thus, the majority of the study participants in the case and control groups had a moderate level of performance before training. There was no statistically significant difference between the performances of the control group before and after training, and the 2 groups (case and control) were homogeneous in terms of performance (P=0.128).

With respect to post-training CPR performance, the results indicated that in the control group, 25% of the participants were weak (25–50 points), 65% were moderate (51–75 points), and 10% were good (76–100 points).And the rate of CPR performance in the case group before training included the following categories:15% were weak (25–50 points), 75% were moderate (51-75 points), and 10% were good (76-100 points). In other words, after the intervention and training, CPR performance was improved in the control group; however, the rate of improvement was higher in the case group, in which the majority had better scores after training. The results of our statistical test showed that the rate of CPR performance had a statistically significant difference in both the case group and the case group after training (P=0.038).

In the control group, the mean score before and after intervention was 55.5 (SD=6.6) and 59.1 (SD=8.7), respectively. In the case group, the corresponding mean score before and after intervention was 60.6 (SD=7.9) and 64.9 (SD=9.1).

Our results showed that using both methods of face-to-face training and video training enhanced CPR performance by comparison with the standard method, which is in line with the findings of a study which showed the positive effect of education on nurses’ performance.^4^ Therefore, the level of CPR performance is negatively impacted by lack of relevant knowledge and it can be improved through appropriate training. In the present study, the rate of improvement in the intervention group, who received training via videos, was statistically significant; nonetheless, face-to-face training had higher efficacy and its impact was more remarkable. 

Thus, it can be concluded that raising awareness about CPR performance among nurses and doctors with the aid of videos can augment the performance score. 
